# Comment on: Evaluation of adjunctive mycophenolate for large vessel giant cell arteritis. Reply

**DOI:** 10.1093/rap/rkab012

**Published:** 2021-02-26

**Authors:** Maira Karabayas

**Affiliations:** 1 Aberdeen Centre for Arthritis and Musculoskeletal Health, University of Aberdeen; 2 Rheumatology Service, NHS Grampian, Aberdeen, UK

Dear E ditor, We thank Drs Mazumder and Mukhtyar for their positive commentary [1] on our paper [2]. It is increasingly recognized that GCA is a heterogeneous disease on the basis not only of clinical phenotypes, but also of immunopathological characteristics. Similar to your thoughts, we propose that future research efforts focus on all these aspects of the disease to map the distinct endotypes and, ultimately, carve the path towards precision medicine in GCA. Although there is emerging evidence to support the distinct subsets within the spectrum of GCA, these observations require validation in multicentre studies, and further research efforts are required to explore in depth the immunobiological drivers of these disease endotypes.

Gribbons *et al.* [3] recently published an analysis of the Diagnostic and Classification Criteria for Vasculitis (DCVAS) data and showed that patients exclusively with large vessel involvement (TA-negative and evidence of large vessel involvement on imaging) differed considerably from patients with isolated temporal artery involvement (TA-positive and evidence of large vessel involvement on imaging) in terms of both their clinical profiles and their demographic characteristics. In parallel, several studies have shown that the expression of specific cytokines and chemokines, at a vascular (tissue) or extravascular level (serum or plasma), are associated with differential risks of cranial ischaemia, indicating that there is an immunological predilection towards the observed clinical phenotype [4].

With regard to treatment approaches for those patients exhibiting both cranial and extracranial features, in theory these should possibly be treated separately. Logically therapies are likely to work differently against different biological profiles and presumably in the future different endotypes will be treated separately. However, in reality, combination immunosuppression can bring greater issues of toxicity; therefore, in practice we think that the most clinically dominating endotype should be targeted in the first instance.

We sympathize with the perennial challenge of accessing therapies off label for patients with rare diseases where clinical trial data are sparse or non-existent. Until recently, there have been no licensed therapies for the indication of large vessel vasculitis. Furthermore, the clinical trials that have been conducted for this condition have been mixed, with no widely recommended therapies other than CSs. Regionally, our health-care system operates a process for approving the use of off-label therapies that is based fundamentally on multidisciplinary team decision making and review of existing scientific evidence/clinical guidelines. In fact, Dr Mukhtyar’s leadership of the original EULAR recommendation for the management of large vessel vasculitis is very supportive in this respect, specifically recommending that an immunosuppressive agent should be considered for use in large vessel vasculitis as adjunctive therapy rather than exposing patients to the long-term toxicities of CSs. We hope that data such as these will support future randomized control trials and, in consequence, make access to targeted therapies universally easier.

We appreciate that there are data to justify the use of MTX in GCA, although effects appear modest [5]. In that study, *n* = 6 patients switched to MTX or tocilizumab owing to significant disease relapse (defined as recurrence of symptoms and/or evidence of disease activity or structural progression on imaging) on mycophenolate derivatives. In our routine clinical practice, patients who fail to tolerate mycophenolate derivatives are often switched to MTX or tocilizumab depending on their clinical phenotype. In fact, MTX is often used as first line in those with significant peripheral arthritis. However, in order to inform the choice of CS-sparing agent, head-to-head trials are required to challenge these agents, ideally within the different disease subsets.

We recognize that symptoms of headache and visual changes were commonly reported amongst patients, as detailed in the manuscript. Owing to the retrospective nature of this study and in the absence of concomitant temporal artery biopsy and PET-CT, it is difficult to say with complete confidence that these symptoms did not genuinely reflect cranial disease. That said, the rationalization of these symptoms relied on the discretion of the clinician and were not felt to be clinically relevant to pursue a temporal artery biopsy. Furthermore, the patient’s perspective of the disease can also act as a bias in this group of patients when often non-classical or unrelated headache and visual disturbance are symptoms commonly over-reported. Finally, the younger age observed in this cohort (mean ± SD 69.4 ± 7.9 years) and high prevalence of constitutional symptoms (86.5% of the cohort) favour the extra-cranial variant, as observed in other studies [3, 4, 6].

It would certainly be interesting to evaluate the use of mycophenolate in cranial GCA. This would also help us to determine whether this disease presentation is biologically distinct.

*Funding*: No specific funding was received from any funding bodies in the public, commercial or not-for-profit sectors to carry out the work described in this manuscript.

*Disclosure statement*: The author has declared no conflicts of interest.
